# Why regulatory indifference towards pharmaceutical pollution of the environment could be a missed opportunity in public health protection. a holistic view

**DOI:** 10.11604/pamj.2017.27.77.10973

**Published:** 2017-06-01

**Authors:** Pakoyo Fadhiru Kamba, Bruhan Kaggwa, Edson Ireeta Munanura, Tom Okurut, Freddy Eric Kitutu

**Affiliations:** 1 Department of Pharmacy, School of Health Sciences, Makerere University, P.O Box 7072, Kampala, Uganda; 2 National Environment Management Authority (NEMA), Plot 17/19/21 Jinja Road, Kampala, Uganda

**Keywords:** Pharmaceutical pollution, drugs, public health protection, disposal, wastewater, antibiotics resistance

## Abstract

The last generation has witnessed bludgeoning of the world's population, a spike in disease burden, and unprecedented levels of pharmaceutical consumption and production. Unfortunately, pharmaceuticals have left their industrial and household confines and leaked into the environment. Pharmaceuticals are now major environmental pollutants, and are ubiquitous in waters and soils. Unlike other environmental contaminants, pharmaceutical pollutants are not yet regulated globally, simply because acute risk assessments show insignificant human health hazard. But the pitfalls of pharmaceutical pollutants extend beyond acute effects to delayed effects from bioaccumulation, amplified effects from drug-drug interactions, exacerbation of drug resistance, and reduction in aquatic and terrestrial food production. Therefore, ignoring pharmaceutical pollutants deprives society of holistic public health protection.

## Commentary

The introduction of modern pharmaceuticals as the primary agents of healing in the early 20^th^century revolutionized health care. To date, at least 1450 distinct active molecular entities (AMEs) with diverse chemistry, pharmacology, and toxicology have made it to the market as medications in over 21,000 pharmaceutical products [1]. Millions more medicinally active compounds have staggered at different stages of drug development. Meanwhile, the last half century has seen the emergence and resurgence of chronic diseases, including HIV/AIDS, cancers, diabetes, cardiovascular diseases and obesity, and a large proportion of the global population is on chronic therapy [2]. Furthermore, the global consumption of drugs for acute conditions has also significantly increased over the years. A recent study of antibiotics consumption in 71 countries representative of all the world's geographic and economic diversity shows that it increased 36% from 54.1 billion standard dosage units (tablets, capsules, ampoules, vials) to 73.6 billion units over the ten year period 2000-2010 [3]. Pharmaceuticals are nowadays mainstays in the world's households. In Europe, a study of 40 households in rural Greece found 557 medications (mean = 8.5, SD = 5.8) spread across 324 active pharmaceutical ingredients (APIs) in their custody [4]. In Asia, a survey in Jordan found an average of 10.9 pharmaceutical items (SD = 5.2) in 219 households [5]. In Sub-Saharan Africa, a survey in Uganda found an average of 6 (SD = 5) drugs in 313 (35%) out of 892 households [6].

Increased drug production and consumption have, however, put enormous pressure on the environment in terms of industrial waste management, disposal of unused pharmaceuticals and disposal of packaging material. With saturation of drug pressure in society, unprecedented levels and diversity of pharmaceuticals have leaked and continue to leak out of their industrial and household confines into the environment. Pharmaceutical contamination has been reported in water systems of all types including fresh and marine water, ground water, surface water, drinking water, and waste water [7]. In the United States, a countrywide survey of 139 water streams found pharmaceuticals, hormones and organic waste water contaminants in 80% of them [8]. In India, heavy contamination with numerous pharmaceuticals has been reported in lakes, rivers, wells, and effluent from wastewater treatment plants for drug manufacturers [9]. In China, pharmaceutical concentrations above the United States EPA safety limit of 1.0 µg/L have been reported in water bodies and effluents from sewage treatment plants [10]. In South Africa, antiretroviral drugs, particularly nevirapine, zidovudine and lopinavir have been detected in rivers and surface water storage dams [11].

If environmental hygiene is to be achieved, pharmaceutical contamination of the environment should be mitigated, the same way other organic contaminants, heavy metals and microorganisms are treated. Unfortunately, there is global indifference towards regulation of pharmaceutical pollutants in the environment, to the extent that even the United States, the United Kingdom and the European Union do not regulate the levels of pharmaceuticals in water bodies [12]. Central to the indifference is the notion that at the subtherapeutic concentrations at which many pharmaceuticals are present in the environment, their biological effects are insignificant. Human health assessments of acute effects of individual pharmaceutical pollutants, which show low hazard potential [13] are cited in support of this view. However, reliance on acute studies ignores the delayed effects for those drugs which bioaccumulate. Additionally, they ignore the potential adverse impact of additive, potentiation and synergistic interactions from simultaneous contamination with multiple pharmaceuticals, which is synonymous with environmental compartments [8, 14]. Furthermore, they seem to ignore the negative socio-economic consequences of pharmaceutical pollutants on crop production and aquaculture [15, 16], as well as the public health ramifications of constant, subtherapeutic antibiotics pressure in maintenance of resistant microbes in the environment [3, 17]. Clearly, rational policy decisions may not be arrived at basing on predicted immediate health effects alone. In this paper, a holistic synthesis of the public health implications of pharmaceutical pollutants in the environment (soil and water bodies) is provided. We demonstrate that medium-to-long human health hazards of pharmaceutical pollutants in the environment are too substantial to ignore, and call for re-evaluation of the longstanding state of indifference.

### The pitfalls of pharmaceutical pollutants in the environment

**Unnecessary exposure to some drugs can be out rightly harmful to humans and other living species**: While the beneficial effect (therapeutic or prophylactic) of a drug is the principal goal of pharmaceutical consumption, drugs often induce undesirable effects in the host. In practice, up to 40% of drug consumers experience adverse drug effects [18]. In fact, because of adverse reactions, some drugs are restricted or contraindicated in certain populations and disease states. For example, many drugs are teratogenic (cause congenital anomalies) and are prohibited in pregnancy, including frequently used ones such non-steroidal anti-inflammatory drugs (NSAIDS), phenytoin, carbamazepine and angiotensin converting enzyme (ACE) inhibitors [19]. Also, hormones and endocrine disrupting agents generally induce biological effects at very minute concentrations [14]. Thirdly, some drugs are allergenic in certain individuals and are contraindicated as such [20]. Meanwhile, drugs are often tolerated differently by different types of organisms, and by different age groups of the same species because pharmacokinetic differences arise from variation in gross and cellular biology [21]. For example, lipophilic drugs which typically have long half lives in humans are rapidly cleared in horses and ruminants due to the presence of more powerful hepatic metabolizing enzymes in the latter [21]. Thus, pharmaceutical doses which are safe in one species are not necessarily tolerated in every other species. Indeed, toxicity and fatalities from pharmaceutical contamination of the environment and food have been reported in fish and birds. In Pakistan, consumption of diclofenac-laced animal carcasses by vultures caused renal failure, massive fatalities and population declines of the birds [22]. In the laboratory, diclofenac at the subtherapeutic concentrations typically detected in the environment is toxic to fish; renal tubular necrosis, hyperplasia and fusion of intestinal villi, and widespread changes in gene expression in fish have been recorded [23]. In France, abdominal swelling, endocrine disruption and reduced population have been recorded in fish exposed to pharmaceutical contaminants in the Dore River [16]. Meanwhile, even at the subtherapeutic concentrations typically consumed through breast milk, drugs such as antineoplastics and radioisotopes can harm neonates and infants, and mothers on these drugs are prohibited from breastfeeding. Indeed, increased risk of congenital malformations, miscarriage and subfertility has been reported in health workers who have had long term minuscule exposure to antineoplastic drugs [24]. Lastly, some drugs are carcinogenic and are a hazard even in minute concentrations; for example, the antineoplastic cisplatin [25].

**Subtherapeutic drug exposures exacerbate drug resistance and treatment failure**: Drug concentrations below the minimum effective dose hardly realize the desired therapeutic effects, and are sometimes detrimental to public health and the environment. This is especially true for antimicrobial agents, where exposure to subtherapeutic concentrations hastens the selection of drug resistant microbes, therapeutic failure, and reduction in available therapeutic choices for infectious diseases [3, 17]. Unsurprisingly, microbiological assessments of environmental samples (soil, water) and waste waters have severally detected antibiotic resistant and often multi-drug resistant bacteria [26].

**Subtherapeutic drug exposures may bioaccumulate into harmful concentrations**: Lipophilic compounds, such as steroid hormones, have tendency to accumulate in tissues and may cause undesirable effects after accumulation in the long term. Populations with inadequately developed or compromised liver and kidney functions, namely, the newborns, infants below two months, prematures, the elderly, and all those with hepatic and renal disorders are at high risk of drug bioaccumulation due to poor biological clearance [27, 28]. Subtherapeutic drug exposure may occur actively through poor adherence to prescribed medications or passively through breast feeding, consumption of contaminated animal products and water, occupational inhalation and contact during pharmaceutical production, and exposure during drug administration to patients. Thus, caution should be taken to taper breastfeeding in vulnerable infants whose mothers are on certain medications.

**Harm may arise from potentiation, synergism and additive effects of multiple contaminants**: It is well known that the biological effects of some drugs are amplified when combined together [14]. Given the prevailing situation where there is simultaneous contamination of water bodies with multiple pharmaceuticals and other organic chemicals, the risk of amplified biological effects of subtherapeutic concentrations of individual contaminants is high. Unsurprisingly, aquatic toxicological studies have reported amplified toxicity of combination pharmaceutical contaminants relative to each alone [29].

**Negative impact of pharmaceuticals on agriculture and aquaculture undermines food security and nutrition**: Plants depend on environmental microbiota both for carbon cycling and nitrogen fixation. When antibiotics and other antimicrobial chemicals infiltrate into soils and agricultural waters at biologically relevant concentrations, the populations of such microbiota can be attenuated [15], undermining nutrient recycling and soil productivity. The concentrations of antibiotics in effluents from some waste water treatment plants is so high and far above human therapeutic concentrations [30] that whole populations of soil microbiota may be decimated if such water is used for agricultural production. Some drugs are also toxic to plants. For example, metronidazole, quinacrine and chloroquine retard the growth of soybean [15]. Lastly, cases of growth defects, morphological deformation, sexual abnormalities and population decline in fish, a major source of protein, have been recorded in water bodies contaminated with pharmaceuticals [16, 23]. If left unmitigated, unscrupulous disposal of drug laced wastes into large water bodies could, therefore, reduce fish populations and exacerbate food insecurity in the vulnerable populations of low income countries.

**Pharmaceutical pollution increases purification cost of drinking water**: Traditional water treatment only reduces, but is unable to rid drinking water of pharmaceuticals [8, 14]. In order to reduce pharmaceutical concentrations to negligible levels, expensive additional procedures such as ozonation, nanofiltration and advanced oxidation are needed to augment the conventional water treatment processes of suspended particle adsorption and biotransformation [14]. Increasing the cost of water treatment would make clean water less accessible and drive the poor towards cheaper but unhygienic water sources, particularly in developing countries. In developing countries, prevention of contamination through regulation of industrial waste management, use of animal manure and disposal of household and hospital medicines and packaging would be a pragmatic option.

Pharmaceuticals have both acute effects and delayed effects, and both direct effects and indirect effects on public health. It is insufficient for policies and decisions on whether to regulate pharmaceutical pollutants in the environment to be premised on predicted acute health effects alone because it falsely assumes that all drugs are safe below their therapeutic doses; that everyone in the population tolerates all drugs; and that antibiotic resistance created by pharmaceuticals in sewage does not transfer to humans. It also ignores the fact that even at subtherapeutic doses, the co-occurrence of pharmaceuticals in water and food presents a risk of untoward drug-drug interaction in the consumer, and that food insecurity directly impedes public health. Ignoring these pitfalls is counterproductive to public health. Therefore, there ought to a rethink of the prevailing regulatory indifference towards pharmaceutical pollution of the environment. Meanwhile, surveillance and research on public health hazards of pharmaceutical pollutants ought to be strengthened, particularly in the developing world where regulatory systems and sanitation infrastructure are weak.

## Competing interests

The authors declare that they have no competing interests.
